# HSPA12B inhibits lipopolysaccharide-induced inflammatory response in human umbilical vein endothelial cells

**DOI:** 10.1111/jcmm.12464

**Published:** 2014-12-24

**Authors:** Jun Wu, Xuehan Li, Lei Huang, Surong Jiang, Fei Tu, Xiaojin Zhang, He Ma, Rongrong Li, Chuanfu Li, Yuehua Li, Zhengnian Ding, Li Liu

**Affiliations:** a Department of Geriatrics, The First Affiliated Hospital of Nanjing Medical UniversityNanjing, China; b Department of Anesthesiology, The First Affiliated Hospital of Nanjing Medical UniversityNanjing, China; c Jiangsu Key Laboratory for Molecular and Medical Biotechnology and College of Life Sciences, Nanjing Normal UniversityNanjing, China; d Department of Surgery, East Tennessee State UniversityJohnson City, TN, USA; e Department of Pathophysiology, Nanjing Medical UniversityNanjing, China

**Keywords:** Heat shock protein A12B, lipopolysaccharide, HUVECs, inflammation, PI3K/Akt signalling

## Abstract

Heat shock protein A12B (HSPA12B) is a newly discovered member of the HSP70 protein family. This study investigated the effects of HSPA12B on lipopolysaccharide (LPS)-induced inflammatory responses in human umbilical vein endothelial cells (HUVECs) and the possible mechanisms involved. A HUVECs inflammatory model was induced by LPS. Overexpression of HSPA12B in HUVECs was achieved by infection with recombinant adenoviruses encoding green fluorescence protein-HSPA12B. Knockdown of HSPA12B was achieved by siRNA technique. Twenty four hours after virus infection or siRNA transfection, HUVECs were stimulated with 1 μg/ml LPS for 4 hrs. Endothelial cell permeability ability was determined by transwell permeability assay. The binding rate of human neutrophilic polymorphonuclear leucocytes (PMN) with HUVECs was examined using myeloperoxidase assay. Cell migrating ability was determined by the wound-healing assay. The mRNA and protein expression levels of interested genes were analyzed by RT-qPCR and Western blot, respectively. The release of cytokines interleukin-6 and tumour necrosis factor-α was measured by ELISA. HSPA12B suppressed LPS-induced HUVEC permeability and reduced PMN adhesion to HUVECs. HSPA12B also inhibited LPS-induced up-regulation of adhesion molecules and inflammatory cytokine expression. By contrast, knockdown of HSPA12B enhanced LPS-induced increases in the expression of adhesion molecules and inflammatory cytokines. Moreover, HSPA12B activated PI3K/Akt signalling pathway and pharmacological inhibition of this pathway by Wortmannin completely abrogated the protection of HSPA12B against inflammatory response in HUVECs. Our results suggest that HSPA12B attenuates LPS-induced inflammatory responses in HUVECs *via* activation of PI3K/Akt signalling pathway.

## Introduction

The inflammatory response of endothelial cells is critical for the pathogenesis of various diseases, such as endotoxic shock, thrombosis, cancer, atherosclerosis and diabetes mellitus 1,2. The cellular inflammatory process is characterized by up-regulation of multiple cytokines and adhesion molecules, and is vital to evoke innate immune response and endothelial dysfunction 3,4. On the other hand, lipopolysaccharide (LPS) is an integral part of the outer membrane of Gram-negative bacteria. Mediated by a toll-like receptor 4 (TLR4) on immune cells, it activates the inhibitors of κB (IκB) kinase complex *via* myeloid differentiation factor 88 (MyD88) dependent pathway, resulting in phosphorylation of IκB and the subsequent activation of the transcription factor nuclear factor-κB (NF-κB), which induces the expression of cytokines such as tumour necrosis factor (TNF)-α, interleukin (IL)-6, ICAM-1, VCAM-1, E-selectin 5–7. Therefore, inhibition of the synthesis or release of these inflammatory mediators might be an effective strategy to prevent inflammatory diseases.

Leucocyte-endothelium interactions are important for the acute inflammatory response during various pathological processes. At the onset of an inflammatory episode, the innate immune system provides the first line of defence when polymorphonuclear neutrophil (PMN) leucocytes rapidly adhere to the vascular endothelium and subsequent transmigrate into the site of inflammation or infection 8. The crucial step between the initial contact and final transmigration of PMN is their tight adhesion to endothelial cells, which is mainly mediated by ICAM-1, VCAM-1 and E-selectin 9. In particular, LPS can stimulate adhesion molecule expression and induce PMN-endothelial cell adhesion.

Heat shock protein A12B (HSPA12B) was first discovered in human atherosclerotic lesions by Han *et al*. 10 and then was identified as the newest member of the HSP70 family. As enriched in endothelial cells, HSPA12B has been demonstrated to play an essential role in the induction of angiogenesis and migration *in vitro*, partially attributable to activation of Akt 11,12. Once Akt is activated, it will limit proinflammatory responses during sepsis/septic shock both *in vitro* and *in vivo* 13–15. Moreover, we reported recently that transgenic mice with overexpression of HSPA12B showed resistance to endotoxin-induced cardiac dysfunction through activation of the PI3K/Akt signalling pathway 16. Based on these previous findings, we have been suggested that HSPA12B may attenuate LPS-induced inflammatory responses *via* PI3K/Akt signalling pathway.

## Materials and methods

### Chemicals

*Escherichia coli* LPS (0111:B4) and Wortmannin (WM) were purchased from Sigma-Aldrich (St. Louis, MO, USA). A Bicinchoninic Acid protein assay kit and supersignal west pico chemiluminescent substrate were obtained from Pierce (Rockford, IL, USA). MTT [3-(4,5-dimethylthiazol-2-yl)-2, 5-diphenyltetrazolium bromide] reagent was from Bio Besic Inc. (Markham, ON, Canada). Foetal bovine serum (FBS) was obtained from Gibco (Grand Island, NY, USA). Antibody information: against α-tubulin, from Sigma-Aldrich; against total Akt and phosphor-Akt (p-Akt), from Cell Signaling Technology (Beverly, MA, USA); against E-selectin, from Abcam (Cambridge, UK); against HSPA12B, ICAM-1, IL-6, TNF-α and VCAM-1, from Santa Cruz Biotechnology (Santa Cruz, CA, USA); against COX-2, from R&D Systems Inc (Minneapolis, MN, USA). ELISA kit was purchased from Yuanye Bio-Technology Co., Ltd (Shanghai, China).

### Cell culture

Human umbilical vein endothelial cells (HUVECs) were isolated from umbilical vein cords of normal pregnancies. Briefly, umbilical veins were rinsed with sterile saline and digested with trypsin (0.25%, Sigma-Aldrich). Harvested cells were cultured in M199 medium (Gibco) supplemented with 10% FBS (Gibco), 100 U/ml penicillin–streptomycin (Invitrogen, Carlsbad, CA, USA) and 0.5 ng/ml fibroblast growth factor-basic (Sigma-Aldrich) in an atmosphere of 5% CO_2_ at 37°C. The medium was refreshed at intervals of 2–3 days at cell confluence and the cells in passage 2–5 were used for experiments 17. The umbilical cords were collected from Department of Obstetrics, the First Affiliated Hospital of Nanjing Medical University. This study has been approved by the ethical committee of the First Affiliated Hospital of Nanjing Medical University (2012-SR-153).

### Construction of recombinant adenoviruses and infection

The human HSPA12B gene consists of 20.4 kb of coding sequence (gene ID: ENSG number 00000132622), 7.3 kb of 5′flanking sequence and promoter, and 3.6 kb of 3′flanking sequence. The HSPA12B gene sequence was amplified by RT-PCR and then cloned into shuttle plasmid pAdTrack-CMV and transformed into *Escherichia coli* BJ5183 cells carrying backbone plasmid pAdEasy-1 to obtain adenovirus plasmid through homologous recombination. The adenovirus vectors encoding HSPA12B were transfected into HEK293 cells. After several rounds of passage in HEK293 cells, the adenovirus vectors were purified using two rounds of cesium chloride density gradient centrifugation. Viral titre was determined by a plaque assay and was expressed as plaque forming units. Purified virus aliquots were stored at −80°C. To overexpress HSPA12B in HUVECs, HUVECs were infected with adenovirus containing human HSPA12B cDNA (containing 3 flags) with a multiplicity of infection (MOI) of 6. An adenoviral vector expressing green fluorescence protein (GFP) was also constructed and used as a control vector (Ad-con). Both constructs were made by Genechem Co., Ltd, Shanghai, China. The overexpression of HSPA12B was verified by immunoblotting for HSPA12B 24 hrs after infection.

### Cell viability assay

Cell viability was determined by a MTT assay. In brief, 1 × 10^4^ HUVECs were seeded in 96-well plates and were infected with Ad-HSPA12B or Ad-con for 24 hrs, respectively. Then 20 μl of 5 mg/ml MTT in PBS solution was added into the media and incubated for 4 hrs. Subsequently, the formazan product was solubilized by the addition of 150 μl of dimethyl sulfoxide. Absorbance was measured at a wavelength of 570 nm using Synergy HT plate reader (Synergy HT; Bio-Tek, Winooski, Vermont, USA) and cellular viability (%) was determined.

### Endothelial permeability assay

Human umbilical vein endothelial cell permeability was determined by measuring FITC-labelled dextran (Sigma-Aldrich) across the monolayer as Hordijk PL described 18. Cells were grown on gelatin-coated Transwell (filter area, 0.33 cm^2^, pore diameter size, 0.4 μm, Corning Costar, Cambridge, MA, USA) membranes. All transfection reagents and chemicals were added to the luminal chamber. FITC-dextran 500000-Conjugate (0.5 mg/ml) was added to the luminal chamber and co-incubated with monolayers at 37°C for 60 min. Samples were removed from both luminal and abluminal chambers for fluorescence determination using a fluorescent plate reader (Synergy HT; Bio-Tek) with an excitation wavelength of 490 nm and an emission wavelength of 520 nm. The readings were converted with the use of a standard curve to FITC-dextran concentration. These concentrations were then used in the following equation to determine the permeability coefficient of FITC-dextran (P):P + ([A]/*t*) × (1/*A*) × (V/[L]) where [A] is abluminal concentration, *t* is time in seconds, *A* is area of membrane in cm^2^, V is volume of abluminal chamber, and [L] is luminal concentration.

### Neutrophil isolation

Human neutrophilic polymorphonuclear leucocytes (PMNs) were isolated from blood obtained through vein-puncture from adult healthy volunteers and collected into syringes containing EDTA. Blood samples were mixed with equal volumes of Polymorphprep™ (Axis-Shield, Oslo, Norway) in a 15-ml centrifuge tube. The samples were centrifuged at 450 × g for 35 min. in a swing-out rotor at 20°C. After centrifugation, two leucocytes bands should be visible. The lower band consisting PMNs was harvested and transferred into a new centrifuge tube with Pasteur pipette. Cells were washed twice with PBS and were resuspended at a final count of 1 × 10^6^ cells/ml. This procedure usually resulted in an approximately 95 ± 5% purity of neutrophils and the cell viability was more than 95% as detected by trypan blue exclusion test 19. This study has been approved by the ethical committee of the First Affiliated Hospital of Nanjing Medical University.

### Endothelial – neutrophil cell adhesion assay

Human umbilical vein endothelial cells were seeded at a concentration of 1 × 10^6^ cells/well into 24-well plates (Corning Costar) and incubated for 24 hrs at 37°C. Cells were infected by Ad-con or Ad-HSPA12B for 24 hrs and then stimulated with 1 μg/ml LPS for 4 hrs. Thereafter, human neutrophils were added at a concentration of 1 × 10^5^ cells/well and co-incubated with HUVECs for 30 min. at 37°C. The monolayers were gently washed twice with PBS and then adhered neutrophils were quantified by a myeloperoxidase (MPO) assay 20. Neutrophils' MPO activities were assessed by measuring hydrogen peroxide-dependent oxidation of 3,3′,5,5′-tetramethylbenzidine (Sigma-Aldrich). PMN adherence was expres-sed as the ratio of adhered neutrophil MPO activity/total neutrophils.

### Monolayer wound-healing migration assay

To measure the migrating ability of HUVECs, a wound-healing assay was carried out. In brief, cells were seeded at a density of 1 × 10^5^ cells/well in 6-well plates in M199 with 10% FBS and were incubated for 24 hrs. The cells were then infected with Ad-HSPA12B or Ad-con for 24 hrs. After infection, cells were allowed to grow to monolayers and interrupted using a 200 μl pipette tip, washed twice with PBS and challenged with 1 μg/ml of LPS for 4 hrs. Cell migration was photographed using a phase contrast microscope (Wetzlar, Hesse-Darmstadt, Germany). The experiments were performed in triplicate.

### ELISA

Human umbilical vein endothelial cells were seeded at a concentration of 1 × 10^5^ cells/well into 24-well plates (Corning Costar) and incubated for 24 hrs at 37°C. Cells were infected by Ad-con or Ad-HSPA12B for 24 hrs and then stimulated with 1 μg/ml LPS for 4 hrs. Subsequently, cell culture supernatants were centrifuged for 10 min. at 1810g (4°C) to remove cell debris and then tests were performed according to the instructions provided by the manufacturer.

### siRNA transfection

Human umbilical vein endothelial cells (5 × 10^5^ cells/well) were plated in six-well plates and allowed to grow overnight. siRNA (200 pmol) and 5 μl Lipofectamine™ 2000 (Invitrogen) were diluted in M199 to a total volume of 250 μl. The diluted siRNA and Lipofectamine™ 2000 were mixed and incubated at room temperature for 20 min. to generate the transfection mixture. The cells were washed with serum-free M199 medium, and the transfection mixture was added to the six-well plates and incubated for 6 hrs. Then six-well plates were cultured in M199 medium supplemented with 10% FBS and observed through a fluorescence microscope after 24 hrs. The inhibitory efficiency of the siRNAs on the expression of HSPA12B mRNA and protein was evaluated by quantitative reverse transcriptase polymerase chain reaction (RT-qPCR, 24 hrs after) and western blot analysis (48 hrs after).

### Western blot

Western blot was performed as described previously 21. Briefly, the cell lysates were prepared, and equal amounts of protein extract were separated by 10% SDS-PAGE and transferred onto Immobilon-P membrane (Millipore Corp., Billerica, Massachusetts, United States). After blocking, membranes were incubated with primary antibody at 4°C overnight followed by incubation with a secondary antibody. The same membranes were also probed with anti-α-Tubulin for loading control. The blots were detected using an ECL kit, and the signals were quantified by scanning densitometry.

### RNA preparation and quantitative real-time PCR (qPCR)

Total RNA was extracted from HUVECs using TRIZOL reagent (Invitrogen) according to the manufacturer's instructions. Two micrograms of total RNA were reverse-transcribed with 200 U Moloney murine leukaemia virus reverse transcriptase (M-MLV, Promega, Madison, WI, USA), and in the presence of 0.5 mmol/l deoxynucleotide triphosphate, 25 U RNase inhibitor, and 0.5 mg N15 random primers, in a total volume of 25 μl. Once cDNA was obtained, qPCR was carried out to quantify gene expression levels. Each reaction was performed in triplicate, in a 25 μl volume of SYBR Green Real-time PCR Master Mix (Toyobo, Osaka, Japan). The PCR programme was designed as follows: 60 sec. at 95°C; followed by 40 cycles of 15 sec. at 95°C; 15 sec. at 60°C; 45 sec. at 72°C; and 5 sec. at 80°C on the plate reader (Rotor Gene-3000; Corbett Research, Sydney, NSW, Australia). All the data were analyzed using β-actin as an internal control. Specific PCR primers (shown in Table1) were designed by Primer 5 software.

**Table 1:** Primer sequences for qPCR. Forward (F) and reverse (R) primers were designed for each gene as specified below

Gene	Forward and reverse primer (5′–3′)
β-Actin	F-ATTGGCAATGAGCGGTTCCGC
	R-CTCCTGCTTGCTGATCC ACATC
TNF-α	F-CCCCAGGGACCTCTCTCTAAT
	R-GGTTT GCTACAACATGGGCTAC
IL-6	F-GGGAAATCGTGGAAATGAGAAA
	R-AAGTGCATCATCGTTGTTCATACA
ICAM-1	F-TACGTGTGCCATGCCTTTAGC
	R-GCCCACAATGACCAGCAGTA
VCAM-1	F-CGAAAG GCCCAGTTGAAGGA
	R-GAGCACGAGAAGCTCAGGAGAAA
HSPA12B	F-ATCGCCACCTTCAAAAGGCAA
	R-CTGTGAGGACCACTTCACGA

### Statistical analysis

Results are expressed as the means ± standard deviation (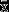 ± SD). Data comparison between groups was performed with one-way anova. Tukey's procedure for multiple range tests was performed. *P* < 0.05 was considered to be significant.

## Results

### LPS induces HUVEC injury in a dose- and time-dependent manner

**Fig 1:**
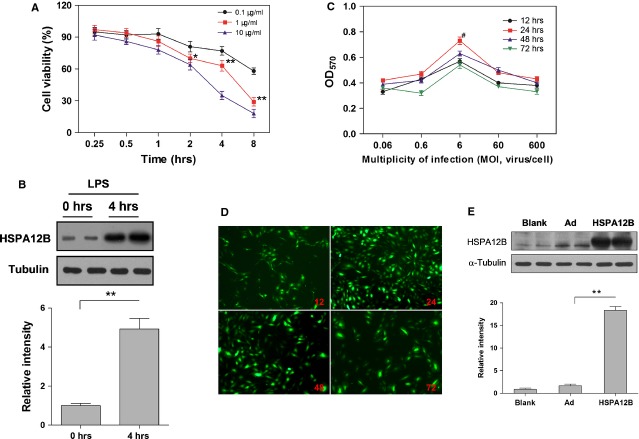
(A) HUVECs viability quantification (%). Cells were treated with LPS (0.1, 1, 10 μg/ml) for 0.25, 0.5, 1, 2, 4 or 8 hrs and the MTT assays were preformed to assess cell viability. *n* + 10 per group. (B) LPS up-regulated HSPA12B expression. HUVECs were stimulated with LPS (1 μg/ml) for 4 hrs. Cells were collected for immunoblotting for HSPA12B. ** *P* < 0.01; *n* + 3 per group. (C) After human HSPA12B gene was transducted by recombinant adenovirus, HUVECs viability was detected by MTT assay. *n* + 3 per group. (D) Efficiency of adenovirus infection determined by GFP expression. (E) Protein expression of HSPA12B was examined by Western blot. * *P* < 0.05, ** *P* < 0.01, *n* + 3 per group.

We incubated HUVECs with LPS at different concentrations for different durations. Cell viability was evaluated by a MTT assay. As shown in Figure1A, LPS decreased cell viability in a time- and dose-dependent manner. Consistently, the percentage of living cells decreased along with the increase in the concentration and incubation time of LPS. Cell viability did not change significantly when HUVECs were exposed to 0.1 or 1 μg/ml LPS for 1 hr. However, HUVECs were almost dead when treated with 1 or 10 μg/ml LPS for 8 hrs. Therefore, we used 1 μg/ml LPS and 4 hrs treatment time in the following experiments.

### LPS up-regulates HSPA12B expression in HUVECs

As shown in Figure1B, treatment with LPS for 4 hrs increased HSPA12B protein levels by 493% in HUVECs, compared with the untreated control HUVECs. The data indicates a possible involvement of HSPA12B in LPS-provoked endothelial inflammation.

### Overexpression of HSPA12B in HUVECs by infection with recombinant adenoviruses encoding GFP-HSPA12B protein

Human umbilical vein endothelial cells were infected by adenoviruses at different MOI index for various durations. MTT assays indicated that cell viability was intact when cells were infected at MOI + 6 for 24 hrs (Fig.1C). Fluorescent microscope examination confirmed the expression of GFP protein in infected HUVECs (Fig.1D). The total HSPA12B protein, which included endogenous HSPA12B (75 kD) and transfected HSPA12B (78 kD, containing 3 flags), was significantly increased by 592.8% in the Ad-HSPA12B group (Fig.1E). These results confirmed the effective expression of exogenous HSPA12B using a recombinant adenovirus expression system.

### Overexpression of HSPA12B inhibits LPS-provoked hyperpermeability

**Fig 2:**
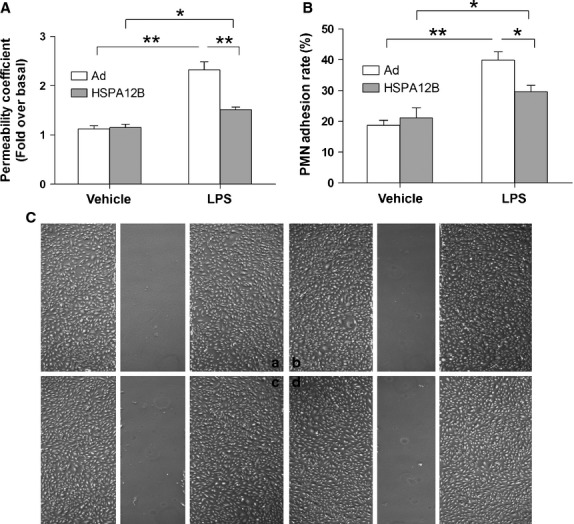
Effects of HSPA12B on LPS-induced endothelial permeability, PMN adhesion, and endothelial cell migration. (A) Permeability. Cells were grown to confluence on transwell membranes and treated with LPS (1 μg/ml) for 4 hrs. FITC-dextran was then added to the luminal chamber, and permeability coefficient of FITC-dextran (P) was calculated after 60 min. *P*-values are expressed as percentages of basal level. *P < 0.05, **P < 0.01. *n* + 3 per group. (B) PMN adhesion. HUVECs were infected with Ad-HSPA12B for 24 hrs, and then were stimulated with 1 μg/ml LPS for 4 hrs, followed by co-incubation with human PMNs for 30 min. PMNs adhesion to HUVECs were measured by assessing MPO activity. *P < 0.05, **P < 0.01. *n* + 3 per group. (C) Endothelial cell migration. Endothelial monolayer was made a wound and administrated with LPS. Endothelial cell migration was examined after stimulation with LPS for 4 hrs. n + 3 per group.

To evaluate the effects of HSPA12B on LPS-induced HUVEC permeability, a transwell assay was used with some modifications 22. As shown in Figure2A, LPS stimulation significantly increased permeability coefficient of FITC-dextran (P) by 2.2-fold, compared with the control group (*P* < 0.01), whereas HSPA12B overexpression significantly suppressed the permeability of HUVECs induced by LPS.

### Overexpression of HSPA12B inhibits PMN adhesion to HUVECs following LPS exposure

As shown in Figure2B, the basal adhesion level of PMN binding to HUVECs was low. However, when the cells were stimulated with LPS for 4 hrs, the rate of PMN binding to HUVECs significantly increased (*P* < 0.01 compared with control). Interestingly, HSPA12B overexpression markedly decreased the adhesion between PMN and HUVECs (*P* < 0.05 compared with LPS-stimulated Ad-con group).

To evaluate the effect of HSPA12B on LPS-induced HUVEC migration, a wound-healing assay was used 23. As shown in Figure2C, only few HUVECs were migrated into wound area following stimulation with LPS for 4 hrs. Moreover, no significant difference of migration was detected between adenovirus group and HSPA12B group that exposed to LPS for 4 hrs.

### Overexpression of HSPA12B attenuates LPS-induced increase in the expression of adhesion molecules and cytokines expression

**Fig 3:**
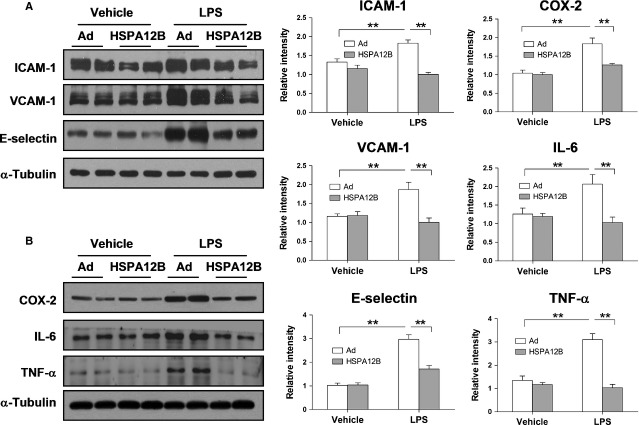
HSPA12B attenuated LPS-induced expression of adhesion molecules and cytokines in HUVECs. HUVECs were infected with Ad-con or Ad-HSPA12B for 24 hrs and then treated with LPS for 4 hrs. The protein levels of ICAM-1, VCAM-1, E-selectin (A), COX-2, IL-6 and TNF-α (B) were detected by Western blot analysis. α-Tubulin was used as the loading control. **P < 0.01, *n* + 3 per group.

**Fig 4:**
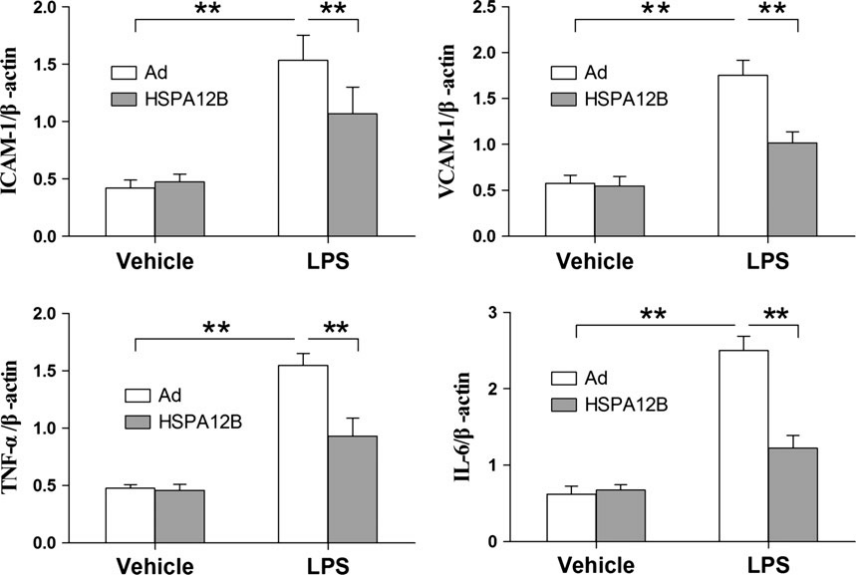
HSPA12B inhibited LPS-induced increases in ICAM-1, VCAM-1, TNF-α and IL-6 in HUVECs. HUVECs were infected with Ad-con or Ad-HSPA12B for 24 hrs and then treated with LPS for 4 hrs. mRNA expression levels of ICAM-1, VCAM-1, TNF-α and IL-6 were quantified by RT-qPCR. β-actin was used as an internal control. *P < 0.05, **P < 0.01, *n* + 3 per group.

As shown in Figure3, LPS increased protein expression of ICAM-1, VCAM-1, E-selectin, COX-2, IL-6 and TNF-α (*P* < 0.01) by western blot analysis, while HSPA12B overexpression inhibited such induction. These findings were confirmed by RT-qPCR analysis, which demonstrated that HSPA12B overexpression attenuated the LPS-induced up-regulation of ICAM-1, VCAM-1, TNF-α and IL-6 mRNA levels in HUVECs (*P* < 0.05 or 0.01, Fig.4). In contrast, Overexpression of HSPA12B markedly decreased such up-regulation.

### Overexpression of HSPA12B reduces IL-6 and TNF-α release from the LPS-stimulated HUVECs

**Fig 5:**
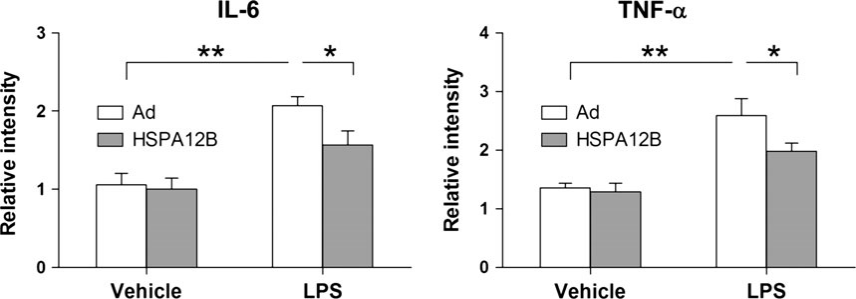
HSPA12B reduced LPS-induced release of IL-6 and TNF-α. HUVECs were infected with Ad-con or Ad-HSPA12B for 24 hrs and then were stimulated with 1 μg/ml LPS for 4 hrs. IL-6 and TNF-α concentration in the culture medium were assayed by ELISA. *P < 0.05, **P < 0.01, *n* + 3 per group.

The release of TNF-α and IL-6 in the LPS-stimulated HUVECs was measured by ELISA. We observed that the contents of IL-6 and TNF-α in culture medium were significantly increased by 1.97- and 2.12-fold after stimulated by LPS in HUVECs (*P* < 0.01). However, the LPS-induced increases in IL-6 and TNF-α release were significantly attenuated by HSPA12B overexpression (*P* < 0.05, Fig.5).

### Knockdown of HSPA12B augments the adhesion of PMN to HUVECs following LPS stimulation

**Fig 6:**
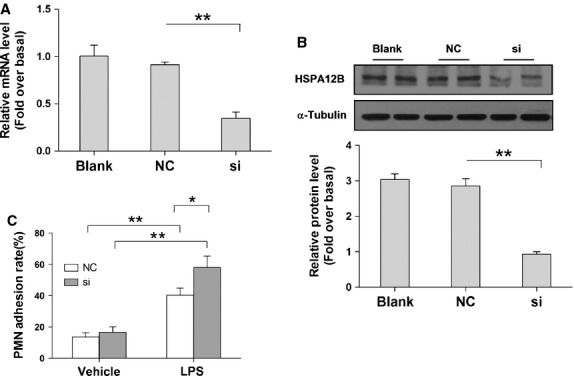
Knockdown of HSPA12B aggravated PMN adhesion. HUVECs were transfected with siRNA to knock down HSPA12B expression. The inhibitory effect of the siRNAs on the expression of HSPA12B mRNA and protein was evaluated by RT-qPCR (A) and western blot analysis (B), respectively. Forty-eight hours after siRNA transfection, cells were stimulated with 1 μg/ml LPS for 4 hrs followed by co-incubation with human PMNs for 30 min. PMNs bound to HUVECs were measured by assessing MPO activity (C). NC + negative control group, si + siRNA group. *P < 0.05, **P < 0.01, *n* + 3 per group.

We next examined the effects of HSPA12B knockdown on the LPS-induced PMN adhesion to HUVCs. Knockdown of HSPA12B was achieved by siRNA transfection. The inhibitory efficiency of siRNA on the expression of HSPA12B mRNA and protein was evaluated by RT-qPCR and western blot analysis. As shown in Figure6A and B, siRNA transfection decreased HSPA12B mRNA level by 65.4% and protein level by 68.75%, respectively, compared with the scramble-transfected (*P* < 0.01). Importantly, the LPS-induced increase in PMN binding to HUVECs was significantly exaggerated by 136.85% by HSPA12B knockdown, compared with that in LPS-treated HUVECs with normal HSPA12B expression (Fig.6C, *P* < 0.05).

### Knockdown of HSPA12B enhances LPS-induced increase in adhesion molecules and cytokines expression

**Fig 7:**
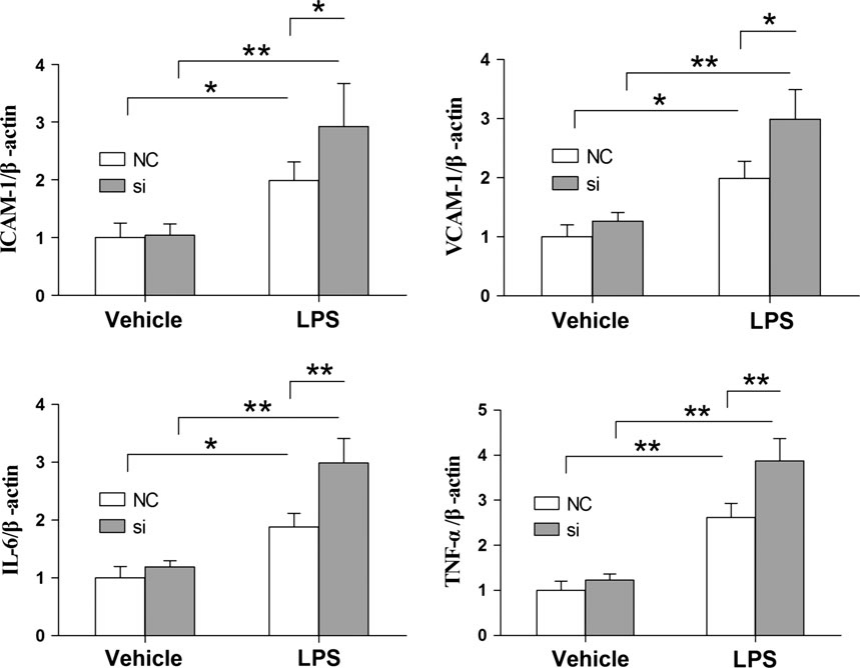
Knockdown of HSPA12B exaggerated LPS-induced increases in ICAM-1, VCAM-1, TNF-α and IL-6 mRNA levels in HUVECs. Forty-eight hours after siRNA transfection, cells were stimulated with 1 μg/ml LPS for 4 hrs. mRNA expression levels of ICAM-1, VCAM-1, TNF-α and IL-6 were quantified by RT-qPCR. β-actin was used as an internal control. NC + negative control group, si + siRNA group. *P < 0.05, **P < 0.01, *n* + 3 per group.

Human umbilical vein endothelial cells were stimulated with LPS at 48 hrs after siRNA transfection. Four hours after LPS stimulation, the mRNA levels of ICAM-1, VCAM-1, IL-6 and TNF-α were analyzed by RT-qPCR. The results demonstrated that the LPS-induced increases in mRNA levels of ICAM-1, VCAM-1, IL-6 and TNF-α were significantly exaggerated by 147.6%, 151%, 161.6% and 148.4%, respectively, compared with the LPS-treated HUVECs with normal HSPA12B (Fig.7, *P* < 0.05).

### Overexpression of HSPA12B prevents the decrease in the phosphorylation levels of Akt in HUVECs with LPS stimulation

**Fig 8:**
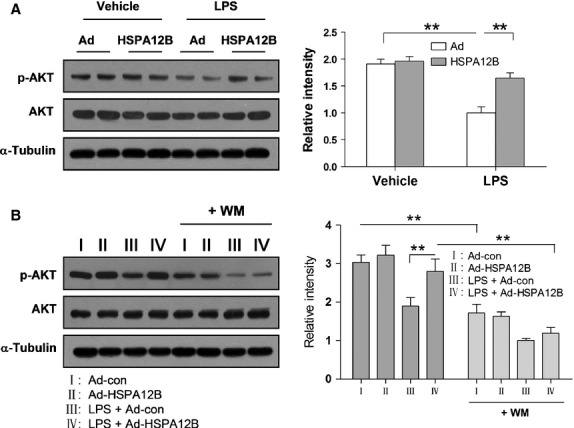
Akt phosphorylation levels. (A) HUVECs were infected with Ad-con or Ad-HSPA12B for 24 hrs and then treated with LPS for 4 hrs. The protein levels of p-Akt and total Akt were detected by western blot analysis. *P < 0.05, **P < 0.01, *n* + 3 per group. (B) Effects of WM on the levels of p-Akt. WM was administrated 30 min. prior to LPS stimulation. The protein levels of p-Akt and total Akt were detected by western blot analysis. *P < 0.05, **P < 0.01, *n* + 3 per group.

Activation of the PI3K/Akt signalling pathway plays an important roles in inflammatory responses 16,24,25, therefore, we examined the effect of HSPA12B on the levels of p-Akt in HUVECs. As shown in Figure8A, LPS treatment for 4 hrs did not cause significant changes in the levels of total Akt, but decreased p-Akt levels by 82.67% compared with untreated control cells (*P* < 0.01). However, in HSPA12B-overexpressed HUVECs, the levels of p-Akt were restored by 56.81% compared with the LPS-treated Ad-con group (*P* < 0.01).

### PI3K inhibition abolishes the inhibitory effects of HSPA12B on LPS-induced inflammatory responses

**Fig 9:**
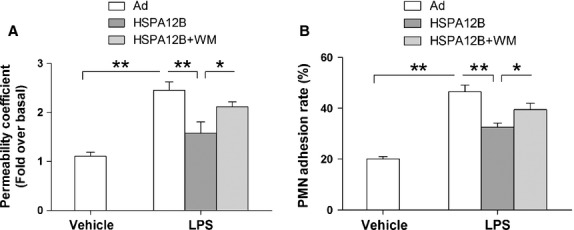
WM abrogated the protective effect of HSPA12B on LPS-induced increases in permeability and PMN adhesion. (A) HUVECs monolayers were treated with LPS for 4 hrs. WM was administrated 30 min. prior to LPS stimulation. Endothelial permeability coefficient of FITC-dextran (P) was examined. (B) HUVECs were treated with LPS for 4 hrs. WM was administrated 30 min. prior to LPS stimulation. PMN adhesion to HUVECs was examined. *P < 0.05, **P < 0.01, *n* + 3 per group.

To determine the role of PI3K/Akt signalling in the protection of HSPA12B against LPS-provoked inflammatory responses, we pretreated HUVECs with WM (a selective inhibitor for PI3K) 30 min. prior to LPS stimulation. As shown in Figure8B, WM significantly decreased the level of p-Akt either in presence or absence of LPS in HUVECs. Importantly, inhibition of Akt activation with WM increased the LPS-induced permeability in HSPA12B-overexpressd HUVECs (Fig.9A). Additionally, WM abrogated the protective effect of HSPA12B on adherence between PMN and HUVEC (Fig.9B). These results suggested that the inhibitory effect of HSPA12B on LPS-induced HUVECs permeability and PMN adherence was mediated by activation of PI3K/Akt signalling pathway.

## Discussion

This study demonstrated that overexpression of HSPA12B inhibited LPS-induced inflammatory responses as indicated by suppression of HUVECs permeability, PMN adhesion to HUVECs and adhesion molecules expression. By contrast, knockdown of HSPA12B aggravated the LPS-induced inflammation in HUVECs. HSPA12B overexpression also markedly prevented the decrease in the levels of phosphorylated Akt in the HUVECs with LPS stimulation. Importantly, PI3K inhibition by WM abrogated HSPA12B-induced protection against LPS-induced inflammation. We conclude that the anti-inflammatory effect of HSPA12B is mediated, at least in part, through activation of the PI3K/Akt signalling pathway.

HSPA12B is a newly discovered HSP and predominantly expressed in endothelial cells, which differs from other HSPs 10–12. Induction of HSPs (*e.g*. Hsp70, Hsp27, αB-crystallin) has been reported to play a protective role in various diseases, such as sepsis, ischaemic heart disease, diabetes, and neurodegeneration 26–28. We have previously demonstrated that overexpression of HSPA12B significantly prevents sepsis-induced cardiac dysfunction and the possible mechanisms include the inhibition of LPS-induced expression of VCAM-1/ICAM-1, and leucocyte infiltration into the myocardium 16. In this study, we observed that overexpression of HSPA12B attenuated LPS-induced inflammatory response in HUVECs, indicating that HSPA12B could improve endothelial dysfunction in inflammatory diseases.

*In vivo* and *in vitro* studies have shown that high-level of LPS exposure can lead to the activation of endothelial cells, secretion of inflammatory cytokines and endothelium injury. Endothelial cell hyperpermeability and the adhesion of circulating PMN to the vascular endothelium are critical steps in the inflammatory response. In this study, cell permeability was induced in LPS-treated HUVECs, however, the enhanced permeability was effectively blocked by overexpression of HSPA12B. In addition, one of the integral parts of vascular inflammation is the PMN adhesion to endothelial cells, which partly attributes to the chemotactic cytokine stimulation, such as ICAM-1, VCAM-1, TNF-α and COX-2 29. As we hypothesized, overexpression of HSPA12B significantly inhibited PMN adhesion to LPS-treated HUVECs.

Numerous studies have shown that LPS increases the expression of ICAM-1, VCAM-1 and E-selectin in both coronary endothelial cells and cardiac myocytes 30,31. In addition to ICAM-1, VCAM-1 and E-selectin, increased activity of TNF-α, IL-6 and COX-2 is positively correlated with initiation and amplification of inflammation 32. Therefore, blockade of either adhesion molecules production or TNF-α/IL-6 activity will reduce leucocyte accumulation and alleviate LPS-induced inflammatory injury. Indeed, we observed that LPS-mediated up-regulation of ICAM-1, VCAM-1, E-selectin, TNF-α, IL-6 and COX-2 was reduced by overexpression of HSPA12B, which is related to the inhibition of endothelial cell permeability and adhesion of PMN to HUVECs.

While increased NF-κB binding activity promotes the production of ICAM-1, VCAM-1, E-selectin, TNF-α, COX-2 and IL-6, activation of the PI3K/Akt pathway negatively regulates NF-κB binding activity 26,33,34. We and others have demonstrated that the activation of PI3K/Akt signalling alleviates endothelial dysfunction caused by LPS-induced inflammation 35,36. Akt is an important kinase downstream of PI3K. To investigate whether PI3K/Akt signalling pathway is involved in HSPA12B-induced endothelial protection, we examined the phosphorylation levels of Akt in LPS-challenged HUVECs. We observed that HSPA12B overexpression significantly restored the decrease in phosphorylated Akt levels in LPS-treated HUVECs. Furthermore, inhibition of PI3K/Akt by WM completely abrogated the protective effects of HSPA12B, which suggested that HSPA12B-evoked protection against LPS-induced inflammatory response was mediated through a PI3K/Akt-dependent mechanism.

In summary, this study has demonstrated that overexpression of HSPA12B inhibited LPS-induced inflammatory responses in HUVECs, and such protection was mediated through a PI3K/Akt-dependent mechanism. Our results suggest that HSPA12B may have a therapeutic potential against endothelial dysfunction.

## References

[b1] Medzhitov R (2008). Origin and physiological roles of inflammation. Nature.

[b2] Meigs JB, Hu FB, Rifai N (2004). Biomarkers of endothelial dysfunction and risk of type 2 diabetes mellitus. JAMA.

[b3] Cines DB, Pollak ES, Buck CA (1998). Endothelial cells in physiology and in the pathophysiology of vascular disorders. Blood.

[b4] Peters K, Unger RE, Brunner J (2003). Molecular basis of endothelial dysfunction in sepsis. Cardiovasc Res.

[b5] Aderem A, Ulevitch RJ (2000). Toll-like receptors in the induction of the innate immune response. Nature.

[b6] Guha M, Mackman N (2001). LPS induction of gene expression in human monocytes. Cell Signalling.

[b7] Yang X, Zhang G, Tang X (2013). Toll-like receptor 4/nuclear factor-κB signaling pathway is involved in ACTG-toxin H-mediated anti-inflammatory effect. Mol Cell Biochem.

[b8] Ploppa A, Schmidt V, Hientz A (2010). Mechanisms of leukocyte distribution during sepsis: an experimental study on the interdependence of cell activation, shear stress and endothelial injury. Crit Care.

[b9] Jiang YR, Chen KJ, Xu YG (2009). Effects of propyl gallate on adhesion of polymorphonuclear leukocytes to human endothelial cells induced by tumor necrosis factor alpha. Chin J Integr Med.

[b10] Han Z, Truong QA, Park S (2003). Two Hsp70 family members expressed in atherosclerotic lesions. Proc Natl Acad Sci USA.

[b11] Steagall RJ, Rusin˜ol AE, Truong QA (2006). HSPA12B is predominantly expressed in endothelial cells and required for angiogenesis. Arterioscler Thromb Vasc Biol.

[b12] Hu G, Tang J, Zhang B (2006). A novel endothelial-specific heat shock protein HspA12B is required in both zebrafish development and endothelial functions *in vitro*. J Cell Sci.

[b13] Zhang WJ, Wei H, Hagen T (2007). Alpha-lipoic acid attenuates LPS-induced inflammatory responses by activating the phosphoinositide 3-kinase/Akt signaling pathway. Proc Natl Acad Sci USA.

[b14] Fukao T, Koyasu S (2003). PI3K and negative regulation of TLR signaling. Trends Immunol.

[b15] Williams DL, Li C, Ha T (2004). Modulation of the phosphoinositide 3-kinase pathway alters innate resistance to polymicrobial sepsis. J Immunol.

[b16] Zhou H, Qian J, Li C (2011). Attenuation of cardiac dysfunction by HSPA12B in endotoxin-induced sepsis in mice through a PI3K-dependent mechanism. Cardiovasc Res.

[b17] Jaffe EA, Nachman RL, Becker CG (1973). Culture of human endothelial cells derived from umbilical veins. Identification by morphologic and immunologic criteria. J Clin Invest.

[b18] Hordijk PL, Anthony E, Mul FP (1999). Vascular endothelial cadherin modulates endothelial monolayer permeability. J Cell Sci.

[b19] Hu Y (2012). Isolation of human and mouse neutrophils *ex vivo* and *in vitro*. Methods Mol Biol.

[b20] Granger DN, Kubes P (1994). The microcirculation and inflammation: modulation of leukocyte–endothelial cell adhesion. J Leukoc Biol.

[b21] Wang X, Zhang X, Cheng Y (2010). Alpha-lipoic acid prevents bupivacaine-induced neuron injury *in vitro* through a PI3K/Akt-dependent mechanism. Neurotoxicology.

[b22] Tinsley JH, Ustinova EE, Xu W (2002). Src-dependent, neutrophil-mediated vascular hyperpermeability and β-catenin modification. Am J Physiol Cell Physiol.

[b23] Shen J, DiCorleto PE (2008). ADP stimulates human endothelial cell migration *via* P2Y1 nucleotide receptor-mediated mitogen-acti-vated protein kinase pathways. Circ Res.

[b24] Xu YQ, Long L, Yan JQ (2013). Simvastatin induces neuroprotection in 6-OHDA-lesioned PC12 *via* the PI3K/AKT/caspase 3 pathway and anti-inflammatory responses. CNS Neurosci Ther.

[b25] Xu CQ, Liu BJ, Wu JF (2010). Icariin attenuates LPS-induced acute inflammatory responses: involvement of PI3K/Akt and NF-kappaB signaling pathway. Eur J Pharmacol.

[b26] You W, Min X, Zhang X (2009). Cardiac-specific expression of heat shock protein 27 attenuated endotoxin-induced cardiac dysfunction and mortality in mice through a PI3K/Akt-dependent mechanism. Shock.

[b27] Sanlorenzo L, Zhao B, Spight D (2004). Heat shock inhibition of lipopolysaccharide-mediated tumor necrosis factor expression is associated with nuclear induction of MKP-1 and inhibition of mitogen-activated protein kinase activation. Crit Care Med.

[b28] Wong HR, Mannix RJ, Rusnak JM (1996). The heat-shock response attenuates lipopolysaccharide-mediated apoptosis in cultured sheep pulmonary artery endothelial cells. Am J Respir Cell Mol Biol.

[b29] Sun DI, Nizamutdinova IT, Kim YM (2008). Bisacurone inhibits adhesion of inflammatory monocytes or cancer cells to endothelial cells through down-regulation of VCAM-1 expression. Int Immunopharmacol.

[b30] Raeburn CD, Calkins CM, Zimmerman MA (2002). ICAM-1 and VCAM-1 mediate endotoxemic myocardial dysfunction independent of neutro-phil accumulation. Am J Physiol Regul Integr Comp Physiol.

[b31] Albelda SM, Smith CW, Ward PA (1994). Adhesion molecules and inflammatory injury. FASEB J.

[b32] Liu XH, Pan LL, Yang HB (2012). Leonurine attenuates lipopolysaccharide induced inflammatory responses in human endothelial cells: involvement of reactive oxygen species and NF-κB pathways. Eur J Pharmacol.

[b33] Tyagi E, Agrawal R, Nath C (2010). Cholinergic protection *via* alpha7 nicotinic acetylcholine receptors and PI3K-Akt pathway in LPS-induced neuroin-flammation. Neurochem Int.

[b34] Li XQ, Cao W, Li T (2009). Amlodipine inhibits TNF-alpha production and attenuates cardiac dysfunction induced by lipopolysac-charide involving PI3K/Akt pathway. Int Immunopharmacol.

[b35] Hyam SR, Lee IA, Gu W (2013). Arctigenin ameliorates inflammation *in vitro* and *in vivo* by inhibiting the PI3K/AKT pathway and polarizing M1 macrophages to M2-like macrophages. Eur J Pharmacol.

[b36] Jung JS, Shin KO, Lee YM (2013). Anti-inflammatory mechanism of exogenous C2 ceramide in lipopolysaccharide-stimulated microglia. Biochim Biophys Acta.

